# Inhibition of eEF-2 kinase sensitizes human nasopharyngeal carcinoma cells to lapatinib-induced apoptosis through the Src and Erk pathways

**DOI:** 10.1186/s12885-016-2853-5

**Published:** 2016-10-19

**Authors:** Lin Liu, PeiYu Huang, ZhiHui Wang, Nan Chen, Con Tang, Zhong Lin, PeiJian Peng

**Affiliations:** 1 Department of Medical Oncology, The Fifth Affiliated Hospital of Sun-Yat-Sen University, 52 Mei Hua Road East, Zhu Hai, 519000 Guangdong Province People’s Republic of China; 2 State Key Laboratory of Oncology in South China; Collaborative Innovation Center for Cancer Medicine; Department of nasopharyngeal carcinoma, Sun Yat-sen University Cancer Center, Guangzhou, China; 3 Department of Surgical Oncology, The Fifth Affiliated Hospital of Sun-Yat-Sen University, Zhu Hai, China

**Keywords:** Nasopharyngeal carcinoma, Lapatinib, eEF-2 kinase, Synergistic effect, Src/Erk signalling pathway

## Abstract

**Background:**

Previous studies have reported that eEF-2 kinase is associated with tumour cell sensitivity to certain therapies. In the present study, we investigated the relationship between eEF-2 kinase and lapatinib, a dual inhibitor of EGFR and HER-2, in nasopharyngeal carcinoma (NPC) cells.

**Methods:**

The effect of treatment on the growth and proliferation of NPC cells was measured by three methods: cell counting, crystal violet staining and colony counting. Apoptosis was evaluated by flow cytometry to determine Annexin V-APC/7-AAD and cleaved PARP levels, and the results were further confirmed by Western blot analysis. The expression of eEF-2 kinase and the impacts of different treatments on different signalling pathways were analysed by Western blot analysis.

**Results:**

The expression of eEF-2 kinase was significantly associated with NPC cell sensitivity to lapatinib. Therefore, suppression of this kinase could increase the cytocidal effect of lapatinib, as well as reduce cell viability and colony formation. Furthermore, inhibition of eEF-2 kinase, by either RNA interference (eEF-2 kinase siRNA or shRNA) or pharmacological inhibition (NH125), enhanced lapatinib-induced apoptosis of NPC cells. The results also showed that lapatinib combined with NH125 had a synergistic effect in NPC cells. In addition, mechanistic analyses revealed that downregulation of the ERK1/2 and Src pathways, but not the AKT pathway, was involved in this sensitizing effect.

**Conclusions:**

The results of this study suggest that targeting eEF-2 kinase may improve the efficacy of therapeutic interventions such as lapatinib in NPC cells.

## Background

Nasopharyngeal carcinoma (NPC) is a rare head and neck cancer found worldwide, but with particular prevalence in southern China and Southeast Asia [1]. The incidence of NPC in high-incidence regions can reach 15–30 per 100,000 [2]. High rates of recurrence and metastasis are the major reasons for poor prognosis. The most successful therapies for NPC are a combination of radiation and chemotherapy; however, the relapse rate for metastatic patients is as high as 82 % [3]. In addition, the side effects of radical radiation severely impact quality of life. Therefore, developing novel therapeutics for NPC, and especially new target agents, is urgent.

The epidermal growth factor receptor (EGFR) signalling pathway is highly correlated with invasion or metastasis of NPC and therefore is indirectly related to poor survival [4]. In endemic areas, both EGFR and HER-2 are co-expressed in approximately 33-87 % of patients with NPC [5, 6], suggesting that EGFRs may be good targets for NPC therapy.

Lapatinib, also known as Tykerb or GW572016, is the first dual tyrosine kinase inhibitor of EGFR and HER-2. Using in vitro NPC models, recent studies have shown that lapatinib also has anti-tumour activity in NPC and inhibits the phosphorylation of both EGFR and HER-2 [1]. Furthermore, a series of preclinical and clinical studies examined the effects of lapatinib in many solid tumours, including breast, lung, hepatocellular and gastric cancers [7–10]. Despite its promising effects, lapatinib has a half maximal inhibitory concentration (IC50) in the micromolar range in insensitive cell lines [1]. Thus, methods to sensitize NPC to lapatinib are currently under investigation.

Eukaryotic elongation factor-2 kinase (eEF-2 kinase), also known as Ca2+/calmodulin-dependent protein kinase III, is a unique enzyme. It participates in the synthesis of various proteins by phosphorylating its only known substrate eEF-2, and it is upregulated in various malignancies [11, 12]. More recently, a number of investigations have reported that eEF-2 kinase can modulate the sensitivity of malignant cells to many agents [13–17].

Since lapatinib has limited cytocidal efficacy, and eEF-2 kinase may regulate the sensitivity of tumour cells, we investigated the effect of eEF-2 kinase inhibition on NPC sensitivity to lapatinib.

## Methods

### Cell lines and culture

Three human NPC cell lines CNE-2, HONE-1 and C666-1 were generously supplied by the State Key Laboratory of Oncology in South China, People’s Republic of China. The cell lines were cultured in RPMI-1640 medium (Gibco BRL Co. Ltd.,USA) supplemented with 10,000 U/ml penicillin and 10 μg/ml streptomycin. For CNE-2 and HONE-1 cells, 10 % foetal bovine serum (FBS) (Gibco) was added, whereas C666-1 required 20 % FBS. Cells were incubated at 37 °C in humidified 5 % carbon dioxide and 95 % air.

### Inhibitors

Lapatinib and NH125 were purchased from Selleck Chemicals (HOU, TX, USA). Stock solutions (1 mM) were prepared using dimethyl sulfoxide (DMSO) and stored at –20 °C. The inhibitors were added to cells using fresh culture medium, ensuring that the concentration of DMSO in the final solution did not exceed 1 % (v/v).

### Cell viability analysis

Briefly, CNE-2, HONE-1 (3.0 × 10^3^/well) and C666-1 cells (1.5 × 10^4^/well) were seeded in 96-well plates and then incubated with different inhibitors at various dilutions for 48 h. Cell viability was assessed using the Cell Counting Kit-8 (CCK-8; Dojindo Co., Japan) following the manufacturer’s instructions. Optical density (OD) was read at 450 nm on an enzyme-linked immunosorbent assay reader (SpectraMax M5; Molecular Devices, Sunnyvale, CA, USA) after 1 to 4 h of incubation. The viability of the DMSO-treated group (control group) was set to 100 %. Viability was calculated as follows: Cell survival rate (%) = (OD value of treatment group/OD value of control group) × 100 %.

### Crystal violet assay

Cells were suspended at a density of 8.0 × 10^4^/well, distributed into six-well plates and treated with lapatinib, NH125 or their combination at the indicated concentrations for 48 h. Following fixation with 4 % paraformaldehyde, the cells were stained with a 1 % crystal violet solution for 20 min and then photographed. The growth-inhibitory effects of the agents were directly proportional to the number of stained cells.

### Colony formation assay

Tumour cells were seeded into six-well plates at a density of 200-400/well and subjected to lapatinib alone or a combination of NH125 and lapatinib. The medium was replaced every 3 days. Cells were stained with 1 % methylene blue for 20 min after 10 days.

### Western blot analysis

Western blot analysis was performed as described previously [18]. The primary antibodies used were eEF2K, phospho-eEF2 (Thr56), cleaved PARP (Asp214) (D64E10), GAPDH, Phospho-p44/42 MAPK (Erk1/2) (Thr202/Tyr204), Phospho-Akt (Ser473) (D9E) and Phospho-Src family (Tyr416) (D49G4). All of the above-mentioned antibodies were obtained from Cell Signaling Technology (Danvers, MA, USA). Anti-hypoxia-inducible factor (HIF)-1α antibody was purchased from BD Biosciences (San Diego, CA, USA). The secondary antibodies were horseradish peroxidase-conjugated goat anti-rabbit or anti-mouse antibodies (1:2000, Santa Cruz, CA, USA).

### Apoptosis detection assay

CNE-2, HONE-1 (8.0 × 10^4^/well) and C666-1 cells (1.6 × 10^5^/well) were seeded into 6-well plates and treated with different inhibitors for 48 h. Apoptosis was then detected by the following procedures.

### Flow cytometry analysis of Annexin V-APC/7-AAD staining

The Annexin V-APC/7-AAD Apoptosis Detection kit (KGA1023-1026, KeyGEN, Nanjing, China) was used for cell staining and flow cytometry (FC500; Beckman Coulter, Brea, CA, USA) following the manufacturer’s instructions. Annexin V-APC-positive cells were considered apoptotic regardless of the 7-AAD status. Experiments were repeated three times, and the results are displayed as histograms.

### Flow cytometric analysis of cleaved PARP

The cells treated above were collected and blocked for 1 h in 5 % bovine serum albumin before staining with a cleaved PARP (Asp214) (D64E10) antibody for 2 h at 37 °C. The cells were then stained with an anti-rabbit IgG (H + L) F(ab′)2 fragment (Alexa Fluor® 555 Conjugate, Life Technologies, LA) antibody for 1 h followed by washing with PBS. After washing, cells were analysed by flow cytometry using the FACScan (BD Biosciences) instrument.

### RNA-mediated gene knockdown

Tumour cells in the logarithmic growth phase were seeded in six-well plates at densities of 8.0 × 10^4^/well (CNE-2 and HONE-1 cells) and 1.6 × 10^5^/well (C666-1 cells). The cells were grown overnight and then transfected with small interfering RNA (siRNA), short hairpin RNA (shRNA) or control RNA, according to the manufacturer's protocols.

### siRNA transfection

eEF-2 kinase siRNA and control siRNA were synthesized by Shanghai Gene-Pharma Co. (Shanghai, China).

### shRNA transfection

Lentivirus-based shRNA targeting eEF-2 kinase and non-targeting shRNA controls were obtained from Genechem Co., Ltd (Shanghai, China).

### Combination index analysis

The combination index (CI) of lapatinib plus NH125 was analysed using CalcuSyn software (Biosoft, Ferguson, MO, USA), which exploits mutually exclusive equations [19] to determine the CI. A CI < 1 indicated synergism, a CI = 1 indicated additivity, and a CI > 1 indicated antagonism.

### Statistical analysis

The experimental results are displayed as means ± standard deviation of the mean. GraphPad Prism 5 software (GraphPad Software, San Diego, CA, USA) was used for the statistical analyses. The Student’s *t* test (two tailed) was used to compare groups, and a *p*-value < 0.05 was considered statistically significant.

## Results

### Inhibition of eEF-2 kinase by NH125 sensitizes NPC cells to lapatinib

Three NPC cell lines, including two poorly differentiated cell lines, CNE-2 and HONE-1, and one Epstein-Barr virus (EBV)-positive cell line, C666-1, were used to investigate the association between lapatinib sensitivity and eEF-2 kinase status. Previous studies have shown that all three cell lines used in this study co-express EGFR and HER-2 to different degrees [1].

**Fig. 1:**
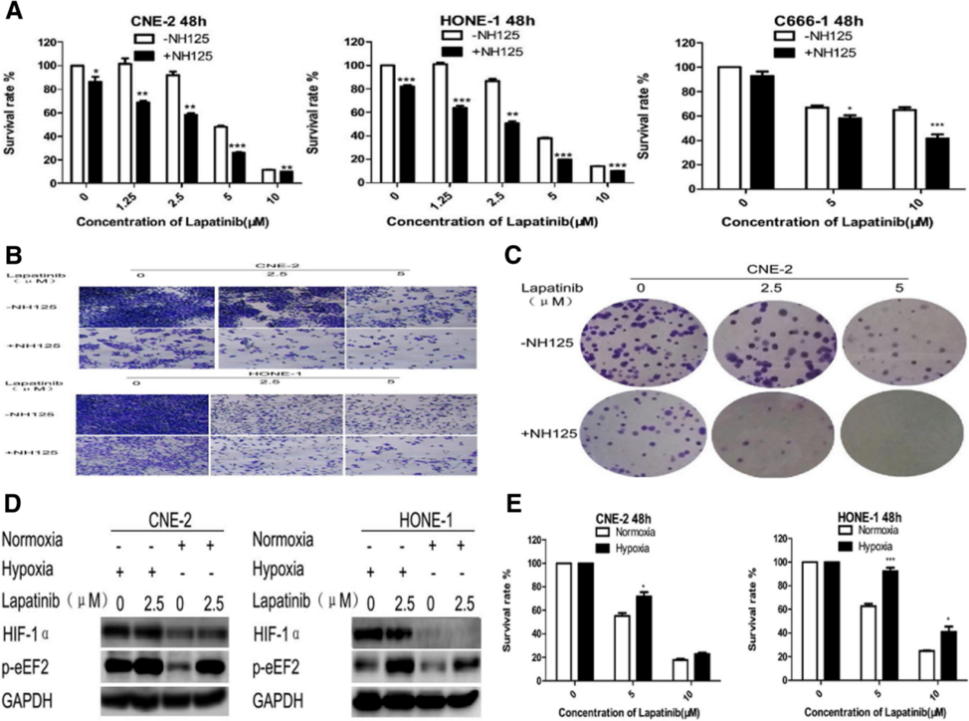
NH125 sensitizes NPC cells to lapatinib. **a**, **b** and **c** NPC cells were treated with lapatinib or DMSO for 48 h in the presence or absence of 0.25 μM NH125. **a** Cell viability was assessed by the CCK-8 assay. Results are expressed as means ± standard deviation. *, *P*<0.05;**, *P*<0.01 and ***, *P*<0.001. **b** Inhibition of proliferation was measured by the crystal violet assay. HONE-1 cells are shown in a representative experiment. **c** Colony formation was measured. CNE-2 cells are shown in a representative experiment **d** and **e** CNE-2 and HONE-1 cells were treated with lapatinib or DMSO under normal or hypoxic (1 % O_2_) conditions for 48 h. **d** HIF-1α and phosphorylated eEF-2 levels were examined by Western blot analysis. GAPDH was used as a loading control. **e** Cell viability was assessed by the CCK-8 assay

The CCK-8 assay was first applied to assess cell viability after 48 h of lapatinib (0-10 μM) treatment with or without 0.25 μmol/L NH125. As shown in Fig. 1a, cell viability was reduced in a dose-dependent manner after lapatinib exposure compared with control cells treated with vehicle DMSO. The cytocidal activity of lapatinib was markedly increased in the cells treated with NH125. A crystal violet assay was used to further validate the above results (Fig. 1b). A 10-day colony formation assay was also performed, and the number of colonies was dramatically reduced by lapatinib combined with NH125 treatment (Fig. 1c).

We next assessed whether eEF-2 kinase activation inhibits the NPC cell response to lapatinib. As shown in Fig. 1d, higher eEF-2 kinase activity (increased phosphorylated eEF-2 levels) was induced by hypoxic conditions. This suggests that hypoxia leads to a reduction in the response to lapatinib, and that eEF-2 kinase activation suppresses the effect of lapatinib in NPC cells (Fig. 1e).

### The eEF-2 kinase inhibitor NH125 enhances lapatinib-induced apoptosis in human NPC cells

**Fig. 2:**
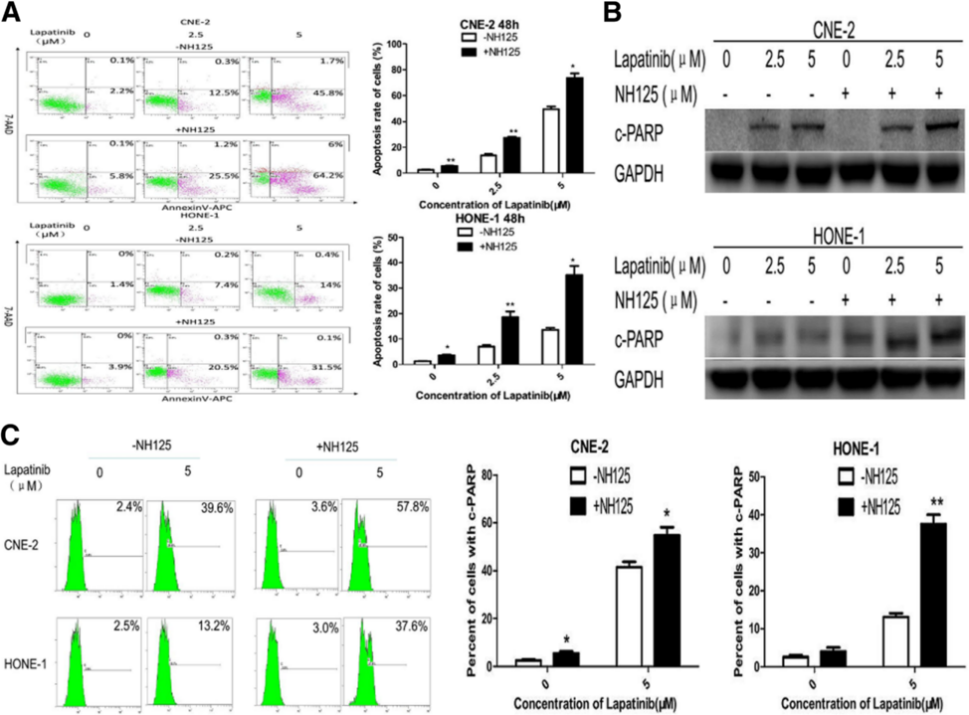
NH125 enhances lapatinib-induced apoptosis in NPC cells. **a**, **b** and **c** CNE-2 and HONE-1 cells were treated with lapatinib (0-5 μM) or DMSO control for 48 h in the presence or absence of 0.25 μM NH125. **a** Annexin V-APC/7-AAD double staining was performed to detect apoptotic activity. **b** Cleaved PARP was examined by Western blot analysis. GAPDH was used as a loading control. **c** Flow cytometry was used to analyse cleaved PARP levels. Results are displayed as histograms. Each bar represents the mean ± standard deviation. *, *P*<0.05;** and *P*<0.01

To confirm and understand better the increased anti-tumour action of lapatinib when combined with NH125, annexin V-APC/7-AAD double staining was used to detect apoptosis after treatment. Lapatinib combined with NH125 significantly increased the population of Annexin V-positive cells and therefore apoptosis (Fig. 2a).

Western blot analysis and flow cytometry were subsequently performed to analyse the levels of cleaved PARP, a marker of apoptosis, in NPC cells in response to treatment. There was a significant increase in the level of cleaved PARP in cells treated with both lapatinib and NH125, suggesting that NH125 increases apoptosis in NPC cell lines (Fig. 2b and c).

### Silencing of eEF-2 kinase by RNA interference increases apoptosis in NPC cells treated with lapatinib

For further verification that eEF-2 kinase has an impact on the sensitivity of NPC cells to lapatinib, we applied RNA interference techniques to inhibit eEF-2 kinase and assessed cell viability and apoptosis after lapatinib treatment.

**Fig. 3:**
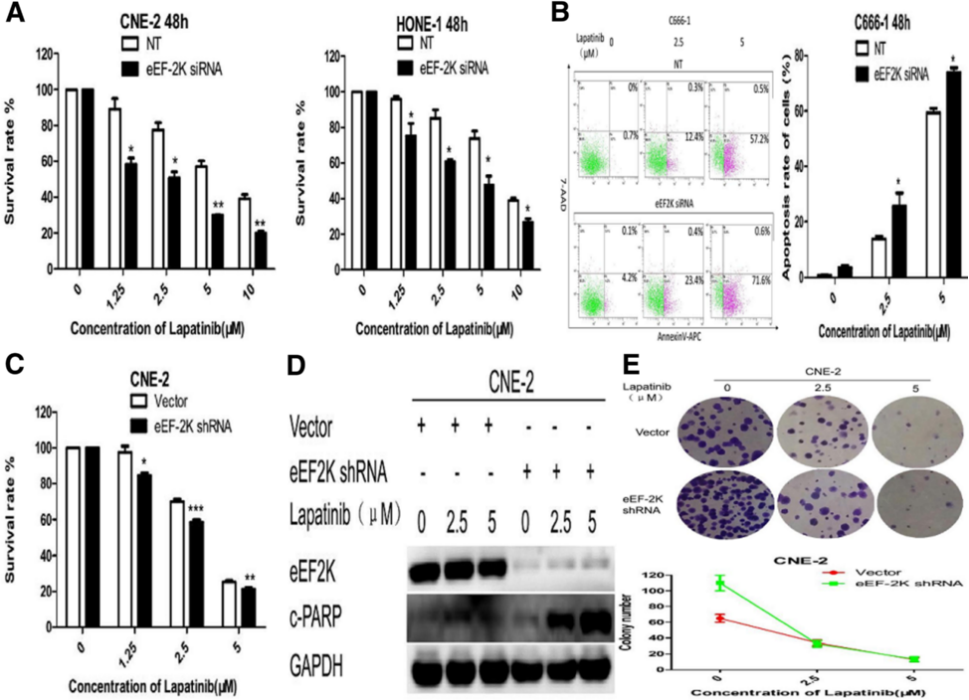
Silencing of eEF-2 kinase expression by RNA interference augments lapatinib-induced apoptosis in NPC cells. **a** and **b** NPC cells were transfected with a non-targeting RNA (NT) or siRNA targeting eEF-2 kinase (eEF-2 K siRNA) followed by treatment with lapatinib or DMSO for 48 h. **a** Cell viability was assessed by the CCK-8 assay. **b** Annexin V-APC/7-AAD double staining was performed to detect apoptotic activity. Results are displayed as histograms. Each bar represents the mean ± standard deviation. *, *P*<0.05 and **, *P*<0.01. **c**, **d** and **e** NPC cells were transfected with an empty vector control (Vector) or a shRNA targeting eEF-2 kinase (eEF-2 K shRNA) followed by treatment with lapatinib or DMSO control for 48 h. **c** Cell viability was assessed by the CCK-8 assay. **d** Cleaved PARP and eEF-2 kinase levels were examined by Western blot analysis. GAPDH was used as a loading control. Results are displayed as histograms. Each bar represents the mean ± standard deviation. *, *P*<0.05;**, *P*<0.01 and ***, *P*<0.001. **e** Colony formation was measured. One representative experiment is shown (CNE-2 cells). Results are displayed as line charts to compare the decreasing trends in colony number

Transfecting NPC cells with an eEF-2 kinase siRNA resulted in a significant decrease in cell viability compared with controls (Fig. 3a). eEF-2 kinase knockdown was also accompanied by an increase in apoptotic activity, as measured by Annexin V-APC/7-AAD double staining (Fig. 3b).

A lentiviral vector carrying a shRNA against eEF-2 kinase was also constructed. The cytotoxicity of lapatinib in NPC cells was greater after shRNA treatment compared with empty vector controls (Fig. 3c). Fig. 3d shows that the shRNA also enhanced apoptotic activity in response to lapatinib. In addition, eEF-2 kinase inhibition decreased colony formation in lapatinib-treated NPC cells (Fig. 3e).

### The synergistic effect of lapatinib and NH125 downregulates the Src/Erk signalling pathway

**Fig. 4:**
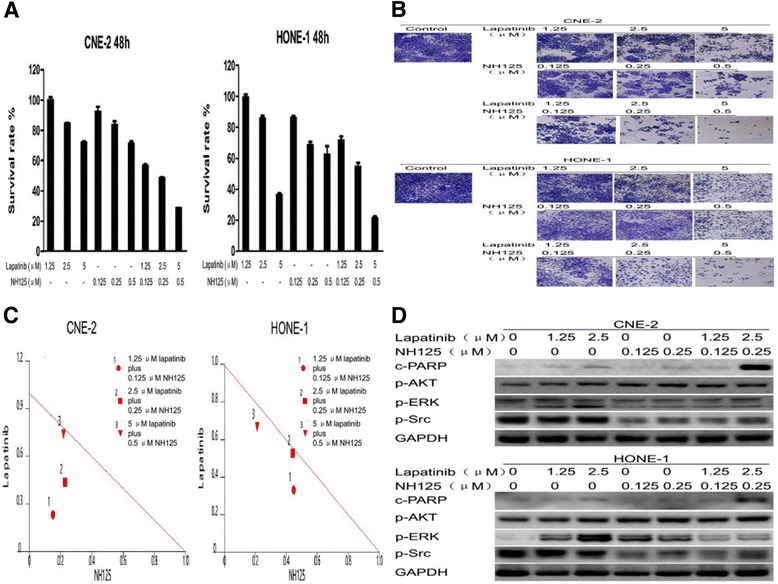
Lapatinib and NH125 exert synergistic effects through the Src and Erk signalling pathways. **a**, **b**, **c** and **d** CNE-2 and HONE-1 cells were treated with lapatinib, NH125 or their combination for 48 h. **a** Cell viability was assessed by the CCK-8 assay. **b** Inhibition of proliferation was measured by the crystal violet assay. **c** IC_50_ isobologram of the lapatinib and NH125 combination treatment. In the isobologram, a plot to the left under the line indicates that the combination is synergistic. **d** Cleaved PARP, phosphorylated AKT, phosphorylated ERK and phosphorylated Src levels were examined by Western blot analysis. GAPDH was used as a loading control

Since inhibition of eEF-2 kinase sensitizes NPC cells to lapatinib, we next evaluated whether lapatinib and eEF-2 kinase inhibition have a synergistic effect. The CCK-8 assay showed that the rate of cell survival was significantly decreased after treatment with lapatinib plus NH125, compared with either treatment alone (Fig. 4a), and the results of crystal violet staining further validated these findings (Fig. 4b). Surprisingly, lapatinib and NH125 had a synergistic effect when treated in combination at a lapatinib:NH125 ratio of 10:1 using lower doses (Fig. 4c).

After this synergistic effect was confirmed, several common signalling pathways were investigated by Western blot analysis. As shown in Fig. 4d, lapatinib alone activated the AKT and ERK pathways in a dose-dependent manner (increased phosphorylated AKT and phosphorylated ERK1/2 levels), but it had no effect on the Src pathway in NPC cells. Furthermore, suppression of eEF-2 kinase activity by NH125 increased the levels of cleaved PARP compared with lapatinib alone. Co-treatment with NH125 and lapatinib decreased Src (decreased phosphorylated Src levels) and ERK (decreased phosphorylated ERK1/2 levels) activities. However, NH125 had no effect on the AKT activity (increased phosphorylated AKT levels) induced by lapatinib.

These results indicate that downregulation of ERK and Src signalling pathways is involved in the synergistic effect between NH125 and lapatinib.

## Discussion

Lapatinib was approved for the treatment of breast cancer due to the correlation between the EGFR and HER-2 signaling and the poor prognosis. Moreover, both EGFR and HER-2 are co-expressed in a high percentage of NPC patients [5, 6]. Therefore, several studies have examined the use of lapatinib in NPC cell lines, but unfortunately the IC_50_ values have ranged widely, from 500 nM to 16 μM [1]. Due to the high IC_50_ values, unsatisfactory therapeutic outcomes have been seen in several studies. In addition, despite good clinical results, lapatinib resistance can result from a variety of mechanisms. Therefore, strategies to augment the anti-tumour efficacy of lapatinib will render this drug more beneficial to patients.

eEF-2 kinase, a critical negative modulator of protein synthesis, has been reported to regulate the sensitivity of cancer cells to several therapeutic drugs, including MK-2206, deoxyglucose, velcade, curcumin, TNF-related apoptosis-inducing ligand and temozolomide [13–17].

Due to the above results, we evaluated whether targeting eEF-2 kinase affects the anti-tumour efficacy of lapatinib in NPC cells. Similar to a previous study [1], phosphorylated EGFR and HER-2 were detected in CNE-2 and HONE-1 cells, but only phosphorylated HER-2 was detected in C666-1 cells. Thus, these three cell lines were used to evaluate the effect of eEF-2 kinase on lapatinib sensitivity.

Similar to previous studies, the results of this study showed that inhibiting eEF-2 kinase through pharmacological or silencing techniques increased the anti-tumour effect of lapatinib by augmenting apoptosis.

Next, we examined whether activating eEF-2 kinase suppresses the cytocidal activity of lapatinib. Hypoxic environments have been reported to induce eEF-2 activity [13], and we further showed that hypoxic conditions decreased the anti-tumour effect of lapatinib by the activation of eEF-2. These results suggest that eEF-2 kinase plays an important role in determining the sensitivity of NPC cells to lapatinib, and that eEF-2 suppression enhances the cytotoxicity of lapatinib. In contrast, the efficacy of lapatinib is reduced when eEF-2 is activated.

We infer that lapatinib and eEF-2 inhibition may have a synergistic effect. Therefore, we investigated the combination effect of NH125 and lapatinib. The results showed that NH125 acts in synergy with lapatinib to increase the cytocidal efficacy. However, the precise molecular mechanism of this effect is unknown. Under environmental or metabolic stress, eEF-2 kinase usually acts as a positive regulator of autophagy [14, 20, 21]. Autophagy can promote both cell survival and cell death depending on the conditions, and it acts to protect cells and tissues from various stresses. Therefore, inhibiting eEF-2 kinase-mediated protective autophagy could enhance cytotoxicity in response to various cancer treatments [13, 17]. Under various stresses, lapatinib has been shown to induce cell death autophagy, which was demonstrated as the type II programmed cell death in hepatocellular carcinoma and chronic myelogenous leukemia K562 cells [22, 23]. Lapatinib also induces type II programmed cell death in NPC cells and we have showed that autophagy is another important mechanism of cell death acting in NPC cells.

To further explore the potential mechanisms, we detected the phosphorylation levels of AKT, ERK and Src, under the condition with or without NH125. There was a significant reduction in the phosphorylation of both ERK and Src, in the treatment with lapatinib and NH125, suggesting that downregulation of ERK and Src signalling is involved in this synergistic effect.

Together, these results suggest that the efficacy of lapatinib in NPC cells can be increased by inhibiting eEF-2 kinase. Therefore, methods to decrease eEF-2 kinase activity should be explored to enhance the efficacy of lapatinib and other cancer treatments.

## Conclusions

Combining lapatinib with NH125 had a synergistic effect in NPC cells by downregulating the Src and Erk signalling pathways and augmenting lapatinib-induced apoptosis. These findings suggest that inhibition of eEF-2 may be a viable method for increasing the efficacy of lapatinib and other cancer therapeutics.
